# Fluorescent collagen hybridizing peptide for quantifying collagen denaturation in cortical bone

**DOI:** 10.1016/j.bonr.2025.101855

**Published:** 2025-06-26

**Authors:** William Woolley, Naomi Chin, S. Michael Yu, Claire Acevedo

**Affiliations:** a Department of Mechanical and Aerospace Engineering, University of California San Diego, La Jolla 92093, CA, USA; b Department of Biomedical Engineering, University of Utah, Salt Lake City 84112, UT, USA

**Keywords:** Confocal, CHP, Mechanical damage, Post-yield, Collagen damage, Denatured collagen

## Abstract

Bone fracture risk is clinically assessed with bone mineral density (BMD); however, individuals with normal BMD also experience fractures, highlighting the need for complementary fracture risk assessment tools. While BMD remains the clinical gold standard, it fails to capture bone quality factors that contribute to fragility. Among these, collagen quality is essential for bone toughness, as it allows collagen to dissipate energy via stretching and uncoiling. When collagen is denatured, it loses its ability to deform, increasing fracture risk. This process is particularly relevant in aging, osteoporosis, and metabolic conditions such as diabetes, yet no clinical methods exist to quantify or localize denatured collagen in mineralized bone. This study introduces Collagen Hybridizing Peptide (CHP) as a tool to quantify denatured collagen in cortical bone. Here, we show that CHP fluorescence correlates strongly with collagen denaturation measured by established trypsin-hydroxyproline assay (r^2^ = 0.99) when applied to mineralized tissue subjected to heat treatment or mechanical loading. Confocal microscopy revealed a 55 % increase in collagen denaturation when tissue strain exceeded the yield point (*p* *<* 0.05). Our findings demonstrate that fluorescent CHP localizes high-strain regions to collagen denaturation on bone fracture surfaces, indicating that collagen damage occurs during post-yield failure. This non-destructive technique offers a powerful tool for assessing collagen quality, with potential applications in osteoporosis, diabetic bone fragility, and aging research. By advancing our ability to evaluate bone quality in cortical bone, R-CHP provides new method to study how denatures collagen affects bone resistance to fracture.

## Highlights

• R-CHP detects denatured collagen in bone without demineralization.
• Confocal R-CHP fluorescence correlates with the well-established T-H assay.
• High staining temperature improves R-CHP penetration in bone.
• Post-yield loading significantly increases collagen denaturation in bone.
• R-CHP localizes collagen damage at bone fracture surfaces.

## 1 Introduction

Predicting fracture risk remains a clinical challenge in several bone fragility diseases (Kadri et al., 2022; Mccreadie and Goldstein, 2000; Ehresman et al., 2021; Poiana and Capatina, 2017; Leslie et al., 2012; Oei et al., 2013). Current clinical assessments, such as DXA-based BMD measurements, fail to explain nearly 50 % of fractures in the aging and osteoporotic populations (Schuit et al., 2004; Mai et al., 2019; Small, 2005). For example, diabetic patients often have normal or even elevated BMD compared to non-diabetic individuals, yet they remain at a significantly higher risk of fractures (Oei et al., 2013; Kao et al., 2003; Tuominen et al., 1999; Schacter and Leslie, 2021). Similarly, 82 % of postmenopausal women who sustained fractures had T-scores above the osteoporosis threshold (−2.5), indicating that most would not have been classified as osteoporotic based on BMD alone. (Siris et al., 2004). These paradoxes emphasize that BMD alone is insufficient for assessing fracture risk and that bone quality factors should be taken into account.

Among bone quality factors, collagen integrity is essential for bone toughness and fracture resistance (Wang et al., 2002; Nyman et al., 2006). Collagen dissipates mechanical energy primarily through its hierarchical and flexible molecular structure. Under mechanical load, collagen fibrils can uncoil, stretch, and slide relative to one another, which delays microcrack formation and distributes stress throughout the matrix (Zimmermann and Ritchie, 2015; Sieverts et al., 2022; Depalle et al., 2016). At the nanoscale, the triple-helical collagen molecules exhibit reversible unfolding and realignment, allowing the matrix to absorb and dissipate energy before structural failure (Zitnay et al., 2020; Zitnay et al., 2017; Szczesny and Elliott, 2014). However, when mechanical loads exceed the tissue's capacity or when collagen integrity is compromised, these molecular mechanisms begin to break down, resulting in irreversible unfolding and denaturation of collagen (Zitnay et al., 2020; Zitnay et al., 2017). In aging and diabetes, this energy dissipation mechanism is further impaired due to the accumulation of advanced glycation end products (AGEs), and matrix stiffening, which limit fibrillar mobility and increase the susceptibility to collagen denaturation (Rosenberg et al., 2024; Rosenberg et al., 2023; Fessel et al., 2014). Because collagen denaturation reflects irreversible molecular damage in the bone matrix, its presence marks regions where the tissue has lost its ability to dissipate energy and resist fracture. In aging and metabolic disease, where BMD measures often fail to explain fragility, denatured collagen may serve as a complementary indicator of compromised bone quality. Therefore, understanding how diseases affect collagen integrity could provide novel insights into fracture risk and guide the development of targeted interventions. Currently, techniques to measure denatured collagen are destructive and lose spatial resolution, making it challenging to investigate how disease-related changes, such as altered remodeling or microdamage accumulation, are linked to specific sites of collagen denaturation. The main established assay (i.e., Trypsin-Hydroxyproline (T-H) assay) consists of digesting the denatured collagen with trypsin and measuring the hydroxyproline content in both damaged and intact collagen to obtain a percentage of denatured collagen (Wang et al., 2000). This T-H assay is well established, but it limits the understanding of the spatial distribution of denatured collagen.

To address this limitation, Collagen Hybridizing Peptide (CHP) offers a novel approach for detecting and quantifying denatured collagen in situ. CHP is a synthetic peptide that selectively binds to unfolded collagen molecules and carries a fluorescent head, enabling direct visualization and localization of collagen damage. Fluorescent CHPs have been successfully developed by Dr. Yu's lab (Li et al., 2013; Hwang et al., 2017) and used in soft tissues to assess collagen denaturation due to mechanical damage, fibrosis, and other pathology (Zitnay et al., 2017; Hwang et al., 2017; Lin et al., 2020; Anderl et al., 2023; Li et al., 2023), but their application in mineralized bone remains limited in part due to challenges with penetration in the mineralized matrix. To date, only two studies have applied CHP to cortical bone, both of which required demineralization prior to staining, limiting its utility for assessing collagen in fully mineralized tissues (Seelemann and Willett, 2022; Yoon et al., 2024). Optimizing CHP staining for cortical bone could establish a new, non-destructive method for evaluating denatured collagen while localizing it. Identifying regions with elevated denatured collagen could reveal local effects of mechanical loading, remodeling activity, or pathological processes on collagen structure. In soft tissues, the usage of CHP in vivo is already achieved (Westenskow et al., 2022; Tao et al., 2023; Subrahmanyam et al., 2023; Arlotta et al., 2020), while in bone, the mineralization still poses difficulties for its application ex vivo.

In this study, we develop a CHP staining protocol for cortical bone ex vivo and validate its effectiveness in detecting collagen denaturation under controlled conditions by comparing it to an established method. By refining CHP-based imaging, we aim to establish a reproducible, spatially resolved, non-destructive approach for assessing collagen integrity in bone. This method would allow for colocalizing regions of denatured collagen with other microstructural features in bone.

## 2 Materials and methods

### 2.1 Study design

**Fig. 1:**
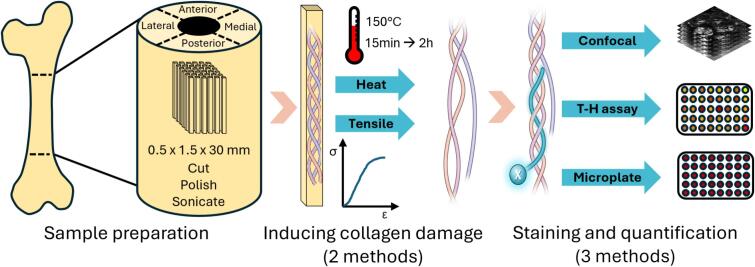
Experimental procedure can be divided into three sections. First, samples are processed from bovine cortical bone. After cutting, polishing, and sonicating matchsticklike samples, the collagen is denatured using two different methods: heat denaturation or mechanical deformation. Heat dematuration is performed at 150 °C. Mechanical loading is performed with a tensile test until failure. Finally, the denatured collagen is stained with R-CHP and quantified using confocal microscopy. The results are compared with established methods, such as the T-H assay.

This study aimed to develop a reproducible and non-destructive method to quantify collagen denaturation in cortical bone samples. Collagen denaturation was induced through controlled heat treatment. CHP fluorescence from confocal imaging was used to quantify the denatured collagen. The accuracy of the method was confirmed by using the well-established T-H assay. The fluorescence measured by confocal was also compared to the bulk fluorescence measured by a destructive assay, which has been shown to be correlated to T-H assay in soft tissues (Lin et al., 2019). Several CHP staining protocols were tested on heat-induced collagen damage to identify optimal staining conditions, including CHP concentration, CHP conjugate, staining temperature, and imaging modalities. The absence of non-specific binding was also assessed using a pre-annealed, triple helical form of CHP. The optimized method was then applied to collagen damaged by mechanical loading to mimic a more physiological condition (Fig. 1).

### 2.2 Specimen preparation

A bovine femur was sectioned into beams with dimensions of 0.5 mm (thickness) x 1.5 mm (width) x 30 mm (length) using a low-speed precision saw (TechCut 5, Allied High Tech Products, USA) under continuous hydration with deionized (DI) water. To achieve smooth surfaces and precise dimensions, beams were sequentially polished using polishing discs with decreasing grit sizes (800 grit to nylon disk) and diamond suspension solution (6 μm to 0.05 μm). Samples were sonicated for 5 min to remove debris and stored in phosphate-buffered saline (PBS, pH 7.4) at 4 °C to maintain hydration before collagen denaturation.

### 2.3 Collagen damage via heat treatment

Samples were exposed to controlled heat treatment in a furnace set to 150 °C for 15 min, 30 min, 1 h, and 2 h. Following heat treatment, beams were immediately rehydrated in PBS at 4 °C for 48 h. This protocol was informed by preliminary studies demonstrating that mineralized collagen denatures at temperatures above 140 °C (Todoh et al., 2009).

### 2.4 Collagen damage via tensile testing

Samples were mechanically damaged using an incremental uniaxial tensile test under displacement control (833 nm/s) with a mechanical tester (Psylotech Incorporated, Evanston, IL, USA). To prevent sliding, the samples were clamped at 0.8 Nm using a torque wrench, and the clamped surfaces were covered with sandpaper to increase friction. The span between the clamps was set to 10 mm. Samples were wrapped in a Kimwipe soaked in PBS to maintain hydration. Preconditioning consisted of five 0.3 % strain cycles. After preconditioning, samples were loaded to 0.4 %, 0.8 %, 1.2 %, and 1.4 % strains, holding each strain level for 90 s. These levels were chosen to cover both pre-yield and post-yield conditions, as the tensile yield point for cortical bone is generally between 0.6 % and 0.8 % strain (Bayraktar et al., 2004). After the test, the load was returned to 0 N, samples were removed, and the clamped ends were cut off to remove the unstrained area. A separate set of samples was tested until failure using the same parameters.

### 2.5 CHP staining

After collagen damage, samples were stained with sulfo-cyanine3 conjugate (R-CHP) (3Helix, Salt Lake City). Since during storage, CHP selfassembles into a trimeric form, and since CHP can bind to denatured collagen only when it is in its monomeric form, trimeric CHP needs to be heated at 80 °C for 10 min to be thermally dissociated into monomers. After being heated, the solution is rapidly cooled down before being applied to the tissue to prevent collagen denaturation due to the temperature. Samples were submerged in 500 μL CHP solution at a 5 μM concentration on a shaker for 24 h. The recommended staining temperature for soft tissues is 4 °C, but to lower the kinetic of self-trimerization, and enhance peptide penetration in cortical bone, different staining temperatures were tested (4 °C, 37 °C, and 50 °C). Based on previous CHP staining experiments, the rinsing protocol was optimized to ensure the complete removal of unbound peptides while preserving signal intensity. Samples were rinsed in phosphate-buffered saline (PBS) for 15 min five times at room temperature. A negative control staining group was done by skipping the thermal dissociation at 80 °C, meaning that CHP at 4 °C (into a trimeric form) was applied to the sample without being able to bind to it.

### 2.6 Confocal imaging

Confocal microscopy (Leica Stellaris 5) was used to image the surface of each sample stained with R-CHP. The objective lens had a 10× magnification. Fluorescence was measured at 548/563 nm excitation/emission for R-CHP. To ensure reproducibility, laser intensity and gain settings were standardized based on preliminary optimization and remained constant across all samples. Autofluorescence correction was applied by thresholding background fluorescence in control samples. To ensure proper focus across non-uniform surfaces, z-stacks were acquired at regular intervals. For penetration analysis, cross-sections were imaged and a ROI ranging from the edge of the bone to the middle axis of the bone was chosen. 3D z-stacks were projected to 2D images using ImageJ (Schneider et al., 2012). Background noise and autofluorescence were removed by applying a threshold that retained the top 5 % of the non-stained sample's histogram intensity. Mean pixel intensity was calculated for surface fluorescence, and penetration depth was assessed by plotting pixel intensity profiles from the edge to the center of the bone cross-section.

### 2.7 Sample demineralization

Following imaging, samples were demineralized in 10 % ethylenediaminetetraacetic acid (EDTA) at 4 °C in the dark for 7 days, with the solution refreshed every 48 h. After demineralization, samples were bisected with a razor blade. One half was used for CHP fluorescence quantification via a microplate assay, and the other half was processed for the T-H assay to measure denatured collagen content.

### 2.8 CHP microplate assay

Samples were placed in 500 μL proteinase K solution (1 mg/mL) for 3 h at 60 °C. Using a microplate reader (SpectraMax Gemini XPS, Molecular Devices, Sunnyvale, CA), the fluorescence of 200 μL duplicates was read at a 548/563 nm excitation/emission for the R-CHP (Lin et al., 2019). After measurement, the two 200 μL duplicates were combined, and the 500 μL solution was used to perform the hydroxyproline assay, measuring the collagen content. The mean fluorescence intensity was normalized to the collagen content.

### 2.9 Trypsin-Hydroxyproline (T-H) assay

Samples were digested in a trypsin solution (0.365 mg trypsin/mg bone) at room temperature for 24 h to isolate denatured collagen. The supernatant (denatured collagen) and remaining bone matrix (intact collagen) were hydrolyzed in 6 M HCl at 110 °C for 18 h. Hydrolyzed products were then oxidized with chloramine-T and *p*-dimethylaminobenzaldehyde (pDAB), producing a colorimetric reaction measured at 561 nm absorbance (Brown et al., 2001). Absorbance values were compared against a standard curve of known hydroxyproline concentrations and converted to collagen by estimating that 14 % hydroxyproline amino acids form a collagen protein. The denatured collagen percentage is then calculated by comparing the denatured collagen content from the supernatant vials to the total collagen content from the supernatant and the intact collagen vial.

### 2.10 Statistical analysis

Statistical analyses were performed using the SciPy package in Python (Virtanen et al., 2020). Group comparisons were conducted for confocal mean pixel intensity, CHP microplate fluorescence intensity, and percent denatured collagen.

**Fig. 2:**
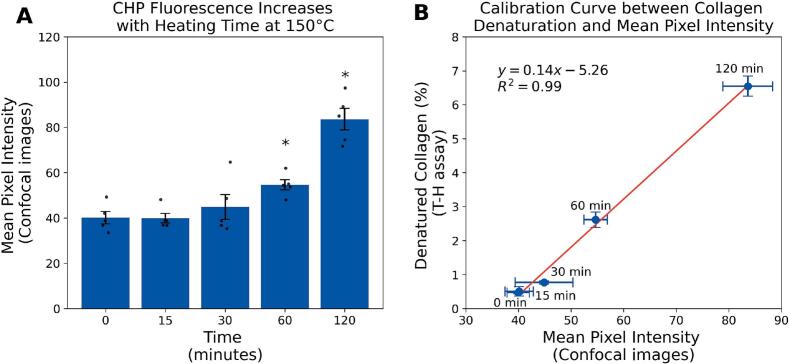
A. Mean R-CHP fluorescence intensity measured by confocal imaging in bone samples heat-treated for 0, 15, 30, 60, or 120 min at 150 °C. Increased fluorescence indicates progressive collagen denaturation. Error bars represent SEM (*n* = 5/group). * denote significant differences from the control group (*p <* 0*.*05, Mann-Whitney *U* test). B. Calibration curve correlating R-CHP mean pixel intensity with percent denatured collagen quantified by the T-H assay. Linear regression shows a strong correlation (*r*^2^ = 0*.*99), enabling estimation of collagen damage from confocal imaging alone.

**Fig. 3:**
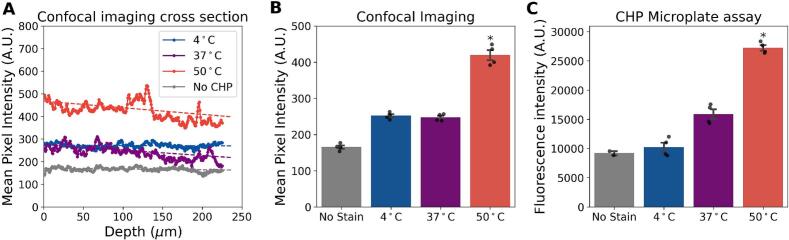
A. Representative depth profile of R-CHP fluorescence from the surface (0 μm) to the center (250 μm) of a bone cross-section stained at different temperatures. Staining at 50 °C led to a stronger and deeper signal compared to 4 °C and 37 °C. B. Average fluorescence intensity measured from confocal imaging across groups confirms increased R-CHP signal at 50 °C. CHP microplate assay validates the improved staining at elevated temperatures across the entire sample. Sample sizes: *n* = 4; *n* = 2 for the non-stained control in the CHP microplate assay. Error bars represent SEM. * indicates significant difference from the control group (*p <* 0*.*05; Mann–Whitney *U* test).

**Fig. 4:**
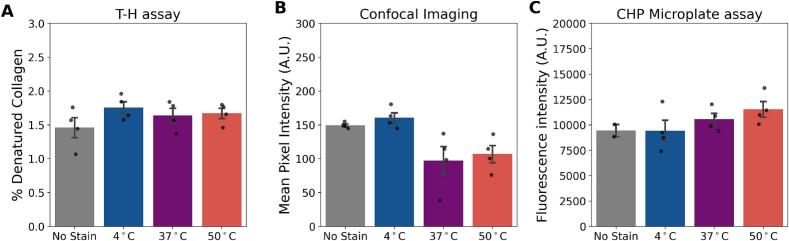
A. The percentage of denatured collagen in non-heated samples shows that the staining temperature does not denature collagen. B. The mean pixel intensity measured from confocal imaging does not show an increase in non-specific binding with the increased temperature of staining. C. CHP microplate assay validates the absence of non-specific binding with the increased staining temperatures across the entire sample. The sample size for each staining temperature group was *n* = 4. Error bars represent the standard error of the mean (SEM).

**Fig. 5:**
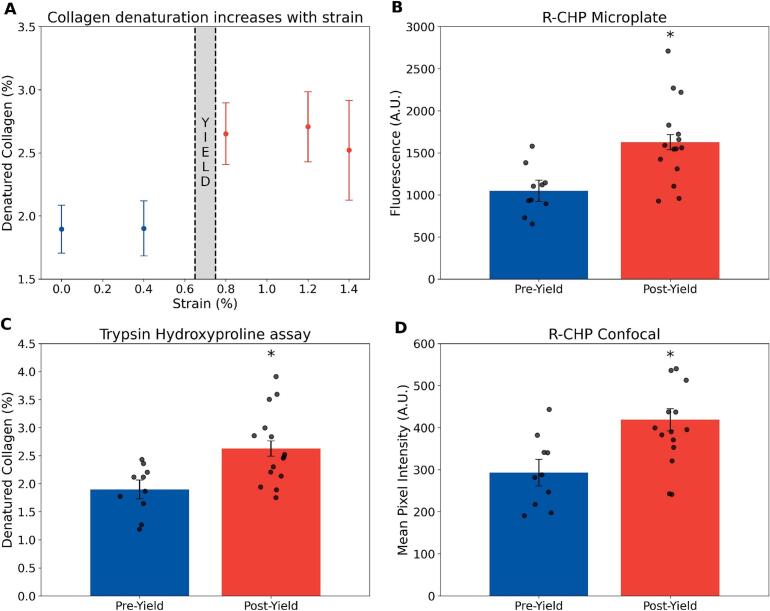
Detection of collagen denaturation after mechanical loading using CHP and T-H assays. A. T-H assay quantification of denatured collagen in samples separated by strain region (pre- and post-yield) (*n* = 5/group). B. Fluorescence intensity measured by CHP microplate assay across pre- and post-yield groups (combined sample groups, *n* = 10–15/group). C. T-H assay results comparing percent denatured collagen across yield phases (combined sample groups, n = 10–15/group). D. Mean pixel intensity from CHP confocal images in pre- and post-yield samples (combined sample groups, n = 10–15/group). Error bars represent the standard error of the mean (SEM). * indicates a significant difference (*p* < 0.05).

Normality and homogeneity of variance were assessed using the ShapiroWilk and Levene's tests, respectively. When assumptions were met and sample sizes were sufficient, parametric tests were used. This was the case for the trypsin-hydroxyproline assay, R-CHP micropalte and R-CHP confocal for the mechanically tested samples (Fig. 5.) For small groups (*n <* 5) or when assumptions were violated, non-parametric tests were applied. This was the case for the trypsin-hydroxyproline assay, R-CHP micropalte and R-CHP confocal in the heat-treated samples (Fig. 2, Fig. 3, and Fig. 4). For the parametric test, we used independent *t*-tests, one-way ANOVA with Tukey post-hoc test, and for the non-parametric test, we used the Mann-Whitney *U* test.

Statistical significance was defined as *p* < 0.05, and data are reported as mean ± standard error of the mean (SEM).

## 3 Results

### 3.1 Heat-induced collagen denaturation is accurately detected with R-CHP in cortical bone

R-CHP mean pixel intensity measured by confocal imaging increased progressively in bone samples heat-treated at 150 °C for 60 and 120 min. Fluorescence intensity increased by 36 % after 60 min (U = 1, *p <* 0*.*05) and by 108 % after 120 min (U = 0, *p <* 0*.*05), indicating greater collagen denaturation (Fig. 2 A). This result was confirmed by the trypsin-hydroxyproline assay, which showed an increase in denatured collagen from 0.51 % in controls to 2.62 %, and 6.55 % in samples heated for 60 min and 120 min, respectively (4-fold and 12-fold increase; *p <* 0*.*05). R-CHP fluorescence strongly correlated with the percent denatured collagen measured by the assay (*r*^2^ = 0.99), validating the accuracy of confocal imaging for estimating collagen damage (Fig. 2 B).

### 3.2 Increased staining temperature improved R-CHP penetration in cortical bone

To assess R-CHP penetration depth, we imaged cross-sections of stained samples and tested whether increasing staining temperature could enhance R-CHP infiltration into the mineralized matrix.

Staining at elevated temperatures improved R-CHP penetration. Samples stained at 50 °C exhibited the highest mean pixel intensity (421 A.U.), compared to those stained at 37 °C (239 A.U.) and 4 °C (252 A.U.) (Fig. 3 B). A depth-dependent decline in fluorescence intensity was observed in samples stained at 50 °C and 37 °C (negative slope). In contrast, samples stained at 4 °C and non-stained controls showed no apparent variation across depth (flat slope) (Fig. 3 A).

These findings were supported by the CHP microplate assay, which quantifies whole-sample fluorescence. Samples stained at 37 °C increased by 73 % without being statistically significant (U = 0, *p* = 0.13) while samples stained at 50 °C showed significant increases in R-CHP signal relative to controls (+196 %, U = 0, *p <* 0*.*05) (Fig. 3 C).

### 3.3 Higher staining temperatures did not increase R-CHP non-specific binding

To confirm that the increased R-CHP signal at higher staining temperatures was not due to additional collagen damage or non-specific binding, we used R-CHP in its triple-helix form, which cannot bind to denatured collagen. Mean pixel intensity did not increase with the different staining temperatures (Fig. 4, B). R-CHP mean pixel intensity was 149 A.U., 160 A.U., 97 A.U., and 106 A.U. for the non-stained, 4 °C, 37 °C, and 50 °C groups, respectively. CHP microplate fluorescence intensity ranged between 9447 A.U. for non-stained samples to 11,528 A.U. for the samples stained at 50 °C (Fig. 4, C). We also confirmed that staining at different temperatures did not increase the level of denatured collagen using the T-H assay. The percentage of denatured collagen was 1.46 %, 1.75 %, 1.63 %, and 1.67 % for the non-stained, 4 °C, 37 °C, and 50 °C groups, respectively (Fig. 4, A).

### 3.4 R-CHP reveals collagen denaturation during post-yield deformation in bone

While heat-induced damage provided a controlled validation of R-CHP sensitivity, we next examined whether CHP confocal imaging could detect mechanically induced collagen denaturation under physiologically relevant conditions. Confocal mean pixel intensity increased significantly in postyield samples compared to pre-yield (+42 %, *p <* 0*.*05) (Fig. 5, D). This was corroborated by the T-H assay, which showed a 38 % increase in denatured collagen post-yield (*p <* 0*.*05; Fig. 5, A, C), and by the CHP microplate assay, which measured a 55 % increase in overall fluorescence in the post-yield region (*p <* 0*.*05; Fig. 5, B). These results confirm that CHP fluorescence effectively reflects collagen denaturation following plastic deformation.

### 3.5 R-CHP reveals localized high collagen denaturation areas on fracture surfaces

**Fig. 6:**
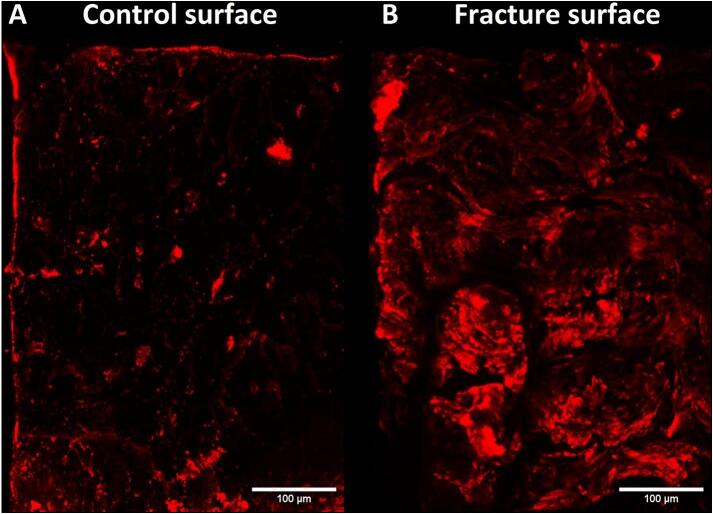
Representative confocal images of cortical bone cross-sections stained with RCHP. A. Control sample cut with a razor blade, showing baseline staining in bone porosity (4.9 % of the ROI). B. Crack surface after tensile failure, revealing extensive regions of denatured collagen along the fracture surface (19.1 % of ROI). Thresholding was applied to retain the top 5 % of intensity values based on the histogram, and the same threshold parameters were used for both images to ensure consistency. The percent area above the threshold was quantified for comparison.

To determine whether R-CHP can localize collagen damage at sites of structural failure, we imaged fracture surfaces following mechanical failure and compared them to razor-cut controls. R-CHP imaging revealed increased collagen denaturation localized at the fracture surface compared to the control sample. Quantification showed that 19.1 % of the region of interest (ROI) was above the fluorescence threshold in the fractured sample, compared to 4.9 % in the control (Fig. 6). In the control, fluorescence was primarily observed in lacunae and canals. Whereas in the mechanically damaged sample, fluorescence displayed concentric layers around canals.

## 4 Discussion

This study establishes R-CHP as a tool for detecting collagen denaturation in mineralized bone ex vivo. We show that R-CHP accurately identifies heat- and mechanically-induced denatured collagen in bone samples without a destructive assay. The strong correlation between R-CHP fluorescence intensity and the T-H assay validated this method as a reliable, non-destructive approach for studying collagen damage in cortical bone.

To develop the use of CHP in bone samples, we denatured collagen via a heat treatment. This method allows to have a diffuse and complete level of collagen denaturation in a bone sample. In this non-physiological method, we showed that R-CHP fluorescence mean pixel intensity correlated strongly with T-H assay results, which serves as a standard. This result is in agreement with similar work performed on tendon where collagen was denatured by heat treatment and CHP fluorescence was compared to the T-H assay (Lin et al., 2019). These results show that CHP can be used not only in soft tissues to measure denatured collagen but also in mineralized tissues. To take into account the difference in the matrix surrounding the collagen, the staining protocol was adapted from soft tissues to mineralized tissue by changing the temperature of staining. Since the mineralized matrix in bone is more dense than that in soft tissues, the penetration of CHP in the sample was a limitation. One approach used when studying collagen in bone is to demineralize the bone before staining, but this approach limits the conclusions that can be drawn from the experiment. Indeed, not being able to relate denatured collagen to microstructural features in bone, such as canals, osteons, lacunae, and canaliculi, is limiting. In this study, we modified the staining temperature to reduce the self-trimerization rate of CHP and allow the penetration of CHP monomers into the sample. The R-CHP mean pixel intensity was higher in the samples stained at 50 °C, showing that staining at a higher temperature improved the R-CHP penetration and staining. This protocol offers a practical solution to the challenge of labeling denatured collagen in mineralized tissues while maintaining sample integrity.

Concerns that high-temperature staining might artificially increase collagen denaturation were addressed by performing T-H assays. We observed no differences in the percentage of denatured collagen between samples stained at different temperatures, confirming that our staining conditions did not induce collagen denaturation. The choice of 50 °C was motivated by the fact that mineralized collagen denatures at higher temperatures than collagen in soft tissues, so at this temperature, the mineralized collagen should not have been damaged (Todoh et al., 2009). Another concern lay in the non-specific binding of RCHP in the sample. We stained samples with CHP in its trimeric form to use it as a negative control, as recommended by the company commercializing CHP (3Helix, and Advanced Biomatrix). CHP in its trimeric form is unable to bind to denatured collagen. We showed that the non-specific binding did not increase with the increased staining temperature. This means that even when the R-CHP penetration is increased, the rinsing steps and the R-CHP specificity allow to have R-CHP to bind only to denatured collagen.

We then applied the improved protocol to mechanically denatured collagen via a tensile test. Since collagen contributes to energy dissipation by deforming plastically (Zimmermann and Ritchie, 2015; Sieverts et al., 2022), we separated the data between the preand post-yield regions to specifically assess damage occurring in the postyield phase, which is closely linked to reduced toughness and increased bone fragility (Sieverts et al., 2022; Wang et al., 2000; Acevedo et al., 2018; Snow et al., 2022). Consistent with collagen's role in energy dissipation via molecular uncoiling and stretching (Depalle et al., 2016), denaturation increased significantly after the yield point and was detected by R-CHP and confirmed with the T-H assay. R-CHP confocal images were able to quantify low levels of collagen denaturation induced mechanically. Our findings on the fracture surfaces confirm that denatured collagen is localized in regions of high strain, such as lamellae around osteons, where energy dissipation prevents catastrophic failure. These results align with previous studies demonstrating that collagen damage occurs during post-yield deformation, further emphasizing its importance in fracture resistance (Sieverts et al., 2022; Lin et al., 2020; Wolfram and Schwiedrzik, 2016). R-CHP provides a new way to spatially map denatured collagen in mechanically loaded bone samples, while also imaging microstructural features.

CHP has never been used in mineralized bone. To date, two studies used CHP in bone but introduced a demineralization step before staining with CHP (Seelemann and Willett, 2022; Yoon et al., 2024). This treatment does not preserve the native structure of mineralized tissues, removing the possibility of relating denatured collagen to microstructural features. Using demineralized bone brings the samples closer to the characteristics of soft tissues. Soft tissue studies have demonstrated that CHP can detect collagen denaturation caused by diseases, mechanical stress, or heat treatment in tendons (Zitnay et al., 2020; Zitnay et al., 2017; Lin et al., 2020; Lin et al., 2019), arteries (Anderl et al., 2023), skin (Hwang et al., 2017), and discs (Dhiman et al., 2024). In soft tissues, CHP has also been applied in vivo to measure retina fibrosis, hepatic fibrosis, breast cancer, and diseased joints in a mouse model (Westenskow et al., 2022; Tao et al., 2023; Subrahmanyam et al., 2023; Arlotta et al., 2020). Although much of this work was based on fluorescent imaging, the latest development of radioactive CHPs is particularly promising for bone application because the signal is not attenuated by the dense mineralized tissue (Ahmad et al., 2025). More research on how to implement CHP in vivo in bone samples would be valuable to relate denatured collagen to other bone quality factors.

While this study offers important insights, there are limitations. Autofluorescence in bone posed challenges. After comparing F-CHP and R-CHP results, we opted to use R-CHP instead of F-CHP due to its reduced overlap with bone autofluorescence. However, exploring far-red fluorophores, such as Cy7, could further minimize interference. The newly developed radioactive CHP developed and used by Ahmad et al. (Ahmad et al., 2025) may enable deeper imaging and reduced soft tissue interference, offering a path toward non-invasive quantification of collagen damage in bone. Despite the improvements made, fluorescence intensity can vary from experiment to experiment, which may affect direct comparisons between experiments. This limits the application of CHP to measure denatured collagen. For now, comparisons of denatured collagen regions can be made between two samples, but if quantitative data are needed, there is a need for a scale or a reference. One way could be to create a calibration curve between the T-H assay and confocal images to estimate the percentage of denatured collagen. But this step would require destroying a piece of the sample. In addition, the sample size for our penetration study was small, limiting the generalization of our results. Still, a large difference was measured between the staining at 50 °C and all the other groups using two different tests. Expanding the sample size would improve the statistical confidence of the results. Regarding crack surface comparison, cutting with a razor blade did not produce a clean fracture and introduced high localized stresses. These stresses may have damaged the collagen, potentially explaining the scattered fluorescence observed.

In conclusion, this work demonstrates the possibility of using CHP in mineralized tissue without a demineralizing step. We showed that R-CHP confocal images can localize regions of denatured collagen and confirmed the accuracy of the staining by comparing the results with the well-established trypsin-hydroxyproline assay. We also showed that R-CHP binds specifically to denatured collagen in bone samples, with minimal non-specific binding. Using CHP in bone would help understand how disease or conditions affect collagen in relation to other microstructural features.

## Declaration of Generative AI and AI-assisted technologies in thewriting process

During the preparation of this work, the author(s) used ChatGPT and Grammarly in order to improve readability and language. After using this tool/service, the author(s) reviewed and edited the content as needed and take(s) full responsibility for the content of the publication.

## CRediT authorship contribution statement

**William Woolley:** Writing – original draft, Visualization, Validation, Methodology, Investigation, Formal analysis, Data curation, Conceptualization. **Naomi Chin:** Visualization, Investigation, Formal analysis. **S. Michael Yu:** Writing – review & editing, Methodology, Conceptualization. **Claire Acevedo:** Writing – review & editing, Writing – original draft, Project administration, Funding acquisition, Conceptualization.

## Declaration of competing interest

S. Michael Yu is a co-founder of 3Helix, Inc., which commercializes collagen hybridizing peptides. All other authors have no professional or financial conflicts of interest to disclose.

## Data Availability

Data will be made available on request.
